# Integrated analysis of the transcriptome, sRNAome, and degradome reveals the network regulating fruit skin coloration in sponge gourd (*Luffa cylindrica*)

**DOI:** 10.1038/s41598-022-07431-w

**Published:** 2022-02-28

**Authors:** Yuyan Sun, Huiqing Zhang, Wenqi Dong, Shengmi He, Shuting Qiao, Xingjiang Qi, Qizan Hu

**Affiliations:** Institute of Vegetables, Zhejiang Academy of Agricultural Sciences, Hangzhou, 310021 China

**Keywords:** Molecular biology, Plant sciences

## Abstract

Sponge gourd fruit skin color is an important quality-related trait because it substantially influences consumer preferences. However, little is known about the miRNAs and genes regulating sponge gourd fruit skin coloration. This study involved an integrated analysis of the transcriptome, sRNAome, and degradome of sponge gourd fruit skins with green skin (GS) and white skin (WS). A total of 4,331 genes were differentially expressed between the GS and WS, with 2,442 down-regulated and 1,889 up-regulated genes in WS. The crucial genes involved in chlorophyll metabolism, chloroplast development, and chloroplast protection were identified (e.g., *HEMA*, *CHLM*, *CRD1*, *POR*, *CAO*, *CLH*, *SGR*, *CAB*, *BEL1-like*, *KNAT*, *ARF*, and peroxidase genes). Additionally, 167 differentially expressed miRNAs were identified, with 70 up-regulated and 97 down-regulated miRNAs in WS. Degradome sequencing identified 125 differentially expressed miRNAs and their 521 differentially expressed target genes. The miR156, miR159, miR166, miR167, miR172, and miR393 targeted the genes involved in chlorophyll metabolism, chloroplast development, and chloroplast protection. Moreover, a flavonoid biosynthesis regulatory network was established involving miR159, miR166, miR169, miR319, miR390, miR396, and their targets *CHS*, *4CL*, *bHLH*, and *MYB*. The qRT-PCR data for the differentially expressed genes were generally consistent with the transcriptome results. Subcellular localization analysis of selected proteins revealed their locations in different cellular compartments, including nucleus, cytoplasm and endoplasmic reticulum. The study findings revealed the important miRNAs, their target genes, and the regulatory network controlling fruit skin coloration in sponge gourd.

## Introduction

Sponge gourd, which is a diploid species with 26 chromosomes (*2n* = *2x* = 26), belongs to the family Cucurbitaceae and genus *Luffa*; it originated in the tropical and subtropical regions of Asia^1,2^. The genus *Luffa* comprises nine species, among which *Luffa cylindrica* (L.) Roem. and *Luffa acutangula* (L.) Roxb. are the main cultivated species worldwide^1,3^. Commercially produced sponge gourd fruits are a rich source of proteins, carbohydrates, vitamins, calcium, phosphorus, iron, and crude fiber^4^. Additionally, sponge gourd fruits contain several compounds with medicinal properties, such as alkaloids, flavonoids, sterols, glycosides, and glycoproteins, making them potentially useful for the pharmacological industry^5–7^. Dried sponge gourd fruits contain large amounts of fiber and can be used as an industrial material (e.g., for generating energy) and as a sponge substitute^4,8,9^. Therefore, sponge gourd is a commercially cultivated crop with nutritional, medicinal, and industrial uses. Accordingly, it should be thoroughly studied.

Sponge gourd fruit skin colors vary greatly (e.g., white, yellowish white, yellowish green, light green, green, and dark green). Fruit skin color is an important quality-related trait for horticultural plants because it considerably influences consumer preferences. To date, many genes related to fruit skin coloration have been reported for horticultural plants. For example, genes controlling the fruit skin colors of cucumber fruits, including white immature fruits (*w* and *w*_*0*_)^10,11^, orange mature fruits (*B*)^12^, and fruits with a yellowish green peel (*ygp*)^13^, have been identified and mapped. Additionally, several genes controlling chloroplast development and chlorophyll biosynthesis have been detected in tomato fruits. The protein encoded by *SlMYB72* directly targets genes involved in the metabolism of chlorophylls, carotenoids, and flavonoids, thereby determining tomato fruit color and ripening^14^. The *GREEN STRIPE* (*GS*) gene, which is a methylated isoform of *TOMATO AGAMOUS-LIKE 1* (*TAGL1)*, reportedly regulates diverse chloroplast developmental processes and carotenoid accumulation in tomato fruit^15^. Moreover, *BEL1-LIKE HOMEODOMAIN 11* (*SlBEL11*)^16^, *KNOTTED1-LIKE HOMEOBOX* (*KNOX*) genes (*TKN2* and *TKN4*)^17^, *ARABIDOPSIS PSEUDO RESPONSE REGULATOR 2-LIKE* (*SlAPRR2-LIKE*)^18^, and the *auxin response factor* (*ARF*) gene *SlARF4*^19,20^ also affect fruit chloroplast development or chlorophyll biosynthesis in tomato. However, the critical genes involved in sponge gourd fruit skin coloration remain unknown.

MicroRNAs (miRNAs) are a class of noncoding short RNAs (19–24 nt) that participate in post-transcriptional regulation by inhibiting translation or cleaving targeted messenger RNAs (mRNAs)^21^. Previous studies revealed a role for several miRNAs in the tissue coloration of horticultural plants^22–26^. Specifically, miR156 and its target *SQUAMOSA PROMOTER BINDING PROTEIN-LIKE* (*SPL*) genes contribute to the regulation of light-induced red peel coloration and anthocyanin accumulation in pear^22^. The MIR156a–*SPL12* module coordinates the accumulation of chlorophylls and anthocyanins during blueberry fruit ripening^23^. An earlier study proved that miR828 regulates phenylpropanoid accumulation by modulating the expression of R2R3-MYB transcription factor-encoding genes and is associated with anthocyanin production in potato^24^. Another study demonstrated that miR858 negatively regulates anthocyanin biosynthesis by repressing the expression of *AaMYBC1* in red kiwifruit^25^. Moreover, the overexpression of bol-miR171b in broccoli results in dark green leaves with increased chlorophyll contents^26^.

Combined multi-omics analyses enabled us to identify several crucial genes and their regulatory network associated with pigment formation in horticultural plants^27,28^. In cucumber, the chlorophyll biosynthetic genes associated with decreased chlorophyll or chloroplast levels in white fruit skin were identified and a predicted anthocyanin biosynthesis regulatory network was established on the basis of an integrated analysis of the metabolome and transcriptome^27^. De novo transcriptome and metabolome analyses of green and purple turnips detected anthocyanins and key genes mediating the difference in root skin pigmentation^28^. Transcriptome, sRNAome, and degradome analyses have been combined to investigate the crucial miRNAs and their target genes as well as the associated network in plants^29–31^. An integrated transcriptome, sRNAome, and degradome examination revealed that miRNAs can mediate tea plant immunity by regulating differentially expressed genes (DEGs) at the post-transcriptional level and miR530b–*ERF96* (encoding ethylene response factor 96) and miRn211–*TLP* (encoding thaumatin-like protein) are important for the responses to gray blight^30^. The high-throughput sequencing of the sRNAome, degradome, and transcriptome identified a novel regulatory pathway involving ethylene–miR164–*NAC* that modulates kiwifruit ripening^31^.

To explore the molecular mechanism underlying sponge gourd fruit skin coloration, we conducted an integrated analysis of the transcriptome, sRNAome, and degradome of two sponge gourd materials with distinct fruit skin colors (i.e., green and white). The key miRNAs, genes, and the network associated with chlorophyll and flavonoid metabolism, chloroplast development, and chloroplast protection were identified. These results provide researchers with valuable information regarding fruit skin coloration and its complex effect on sponge gourd fruit quality.

## Materials and methods

### Plant materials

Two sponge gourd inbred lines, WS and GS, with distinct fruit skin colors, were used in this study. Mature WS fruits had a white peel with a green base, whereas mature GS fruits had a green peel and base. These materials were obtained and preserved in our team, the Specialty Vegetable Breeding Laboratory, Institute of Vegetables, Zhejiang Academy of Agricultural Sciences. Plant materials were planted at the Haining Innovation Base of Zhejiang Academy of Agricultural Sciences, under standard field management. The fruit skin samples were collected from the middle part of the fruits at the mature fruit stage, then were immediately frozen in liquid nitrogen and stored at − 80 °C. Three biological replicates were analyzed for both materials. The collection of plant material complied with the relevant institutional, national, and international guidelines and legislation.

### Measurement of chlorophyll contents and data analyses

For both GS and WS lines, approximately 1.0 g fruit skin samples per replicate were weighed and ground into powder in liquid nitrogen. The ground material was added to 10 mL ethanol and incubated at room temperature until the material turned completely white. The samples were passed through filter paper. The filtrate was topped up with ethanol for a final volume of 30 mL. The absorbance of the samples was measured at 649 nm (A649) and 665 nm (A665) using a spectrophotometer, with ethanol as the blank control. Three replicates of chlorophyll contents were analyzed for both GS and WS lines. The chlorophyll contents were calculated and the data were analyzed by T-test using SAS 8.0 software with *p* < 0.01.

### Transmission electron microscopy analysis

To observe the chloroplast ultrastructure of the fruit skin, WS and GS fruit skin tissues were excised using a sterile razor blade and then immediately fixed in 2.5% (v/v) glutaraldehyde solution. The samples were subsequently rinsed with 0.1 M phosphate buffered solution (PBS) buffer, fixed in 1.0% (v/v) osmium tetroxide, rinsed with 0.1 M PBS buffer, dehydrated using graded ethanol, embedded using the Spurr kit, and polymerized at 70 °C. Ultrathin sections cut using a Leica UC6 ultra-microtome (Leica, Germany) were stained with uranyl acetate for 15 min and then with lead citrate for 5 min at room temperature. Chloroplasts were examined using the H7650 microscope (Hitachi, Tokyo, Japan).

### mRNA library construction and sequencing

Total RNA was isolated and purified using the TRIzol reagent (Invitrogen, Carlsbad, CA, USA) following the manufacturer’s recommended procedure. Poly-(A) RNA was purified from 1 μg total RNA using Dynabeads Oligo-(dT)25-61005 (Thermo Fisher, CA, USA), with two rounds of purification. The poly-(A) RNA was fragmented at 94 °C using the Magnesium RNA Fragmentation Module (NEB, USA). The RNA fragments were reverse transcribed to cDNA using SuperScript II Reverse Transcriptase (Invitrogen). The first-strand cDNA was then used to synthesize U-labeled second-stranded cDNA. An A-base was then added to the blunt ends of each strand to prepare them for the ligation to the index adapters. Single- or dual-index adapters were ligated to the fragments before the size selection step was performed using AMPureXP beads. After the heat-labile UDG enzyme (NEB, USA) treatment of the U-labeled cDNA, the ligated products were amplified by PCR. The average insert size for the final cDNA library was 300 ± 50 bp. Finally, the cDNA library was sequenced (2 × 150-bp paired-end sequencing) on the Illumina NovaSeq 6000 system (LC-Bio, Hangzhou, China) following the vendor’s recommended protocol.

### Sequence mapping and bioinformatic analysis of mRNAs

The Cutadapt software^32^ was used to remove reads with adapters. After eliminating the low-quality reads and the reads with undetermined bases, the HISAT2 software^33^ was used to map the remaining reads to the *Luffa cylindrica* genome^34^. The mapped reads of each sample were assembled using the default parameters of StringTie^35^. The transcriptomes of all samples were merged to construct a comprehensive transcriptome using the gffcompare software (https://github.com/gpertea/gffcompare).

After generating the final transcriptome, StringTie^35^ and ballgown^36^ were used to estimate the expression levels of all genes, which were determined in terms of fragments per kilobase of transcript per million fragments mapped (FPKM) values^37^. The differentially expressed genes (DEGs) were identified using DESeq2, with the following criteria: fold-change > 2 or < 0.5 and *p* < 0.05^38^. The functions of the DEGs were determined on the basis of GO enrichment^39^ and KEGG enrichment^40^ analyses. Heatmap of DEGs bioinformatic analysis was performed using the OmicStudio tools at https://www.omicstudio.cn/tool.

### sRNA library construction and sequencing

TruSeq Small RNA Sample Prep Kits (Illumina, San Diego, USA) were used to prepare sRNA sequencing libraries, which were then sequenced (1 × 50-bp single-end sequencing) on the Illumina HiSeq 2500 system (LC-Bio).

### sRNA sequence analysis

Raw reads were analyzed using the in-house program ACGT101-miR (LC Sciences, Houston, TX, USA) to remove adapter dimers, junk, low-complexity sequences, common RNA families (e.g., rRNAs, tRNAs, snRNAs, and snoRNAs), and repeats. Unique sequences 18–25 nt long were mapped to precursors from specific species in the miRBase 22.0 database^41^ to identify known miRNAs and novel 3p- and 5p-derived miRNAs. Length variations at the 3′ and 5′ ends and one mismatch within the sequence were allowed for the alignment. The unique sequences mapped to the hairpin arms of mature miRNAs from specific species were designated as known miRNAs. The unique sequences mapped to the other arm of known precursor hairpins opposite of the annotated mature miRNA-containing arm were designated as novel 5p- or 3p-derived miRNA candidates. The remaining sequences were mapped to precursor hairpins from other selected species (i.e., specific species were excluded) in the miRBase 22.0 database via a BLAST search. The mapped pre-miRNAs were used as queries in a BLAST search of the genomes from specific species to determine their genomic locations. These sequences were defined as known miRNAs. The unmapped sequences served as queries for a BLAST search of specific genomes. The hairpin RNA structures containing these sequences were predicted according to the flanking 120-nt sequences using the RNAfold software.

### Analysis of differentially expressed miRNAs (DE-miRNAs)

The DE-miRNAs identified on the basis of normalized deep-sequencing read counts were analyzed using the T-test. The significance threshold was set at 0.05.

### cDNA library construction for degradome sequencing

Total RNA was isolated and purified using the TRIzol reagent (Invitrogen) following the manufacturer’s recommended protocol. Poly-(A) RNA was purified from the total RNA (20 µg) using oligo-(dT) magnetic beads. The 5′ adapters were ligated to the 5′ end of the 3′ mRNA cleavage products using RNA ligase. First-strand cDNA was synthesized by reverse transcription involving a 3′-adapter random primer prior to the size selection step performed using AMPureXP beads. The cDNA was amplified by PCR. The average insert size for the final cDNA library was 200–400 bp. Finally, the cDNA library was sequenced (1 × 50-bp single-end sequencing) on the Illumina HiSeq 2500 system (LC-Bio) following the vendor’s recommended procedure.

### Degradome sequencing data processing and target identification

The raw data were processed to obtain sequences suitable for the subsequent analysis. The sequences were aligned with the cDNA sequences in the database of sequenced species to produce the degradome density file. The mRNA sequences of the target genes paired with the sRNA sequences were predicted using the cleavage site prediction software CleaveLand4:GSTAr. The predicted target genes corresponding to miRNAs were combined with the mRNAs in the degradome density file to identify the common mRNAs, which were designated as the miRNA target genes.

### qRT-PCR validation of selected DEGs

Several DEGs involved in fruit skin coloration were selected to verify their expression patterns. Total RNA was reverse transcribed into cDNA using the TransScript One-Step gDNA Removal and cDNA Synthesis SuperMix (TransGen Biotech, Beijing, China). The qRT-PCR analysis was completed using the StepOne Real-Time PCR System (ABI, Foster City, CA, USA) with the following program: 95 °C for 30 s; 40 cycles of 95 °C for 5 s, 55 °C for 15 s, and 72 °C for 10 s. Each experiment was performed in three biological replicates. The relative gene expressions were estimated on the basis of the threshold cycles according to the 2^−∆∆CT^ method^42^. Statistical analysis of relative gene expression were carried out using WPS Excel and SAS 8.0 software. The pearson correlation coefficients analysis of RNA-Seq and qRT-PCR results were performed by SAS 8.0 software. The primers used in this study are listed in Table S1.

### Subcellular localization analyses of selected proteins

The CDS sequences of ten selected proteins from GS or WS were amplified and cloned into the pFGC-eGFP plasmid using One-step Fusion Cloning Mix (Toroivd, Japan) using gene-specific primer with *Bam HI* cleavage site (Table S2). These recombinant plasmids were transformed into *Agrobacteriumt tumefaciens* GV3101 and transiently expressed in tobacco leaf cells. Images were acquired at 48 h using a Leica DMLE camera (Leica, Wetzlar, Germany).

### Ethical approval

This article does not contain any studies with human participants or animals performed by any of the authors.

## Results

### Phenotypes, chloroplast ultrastructures, and chlorophyll contents of WS and GS

**Figure 1:**
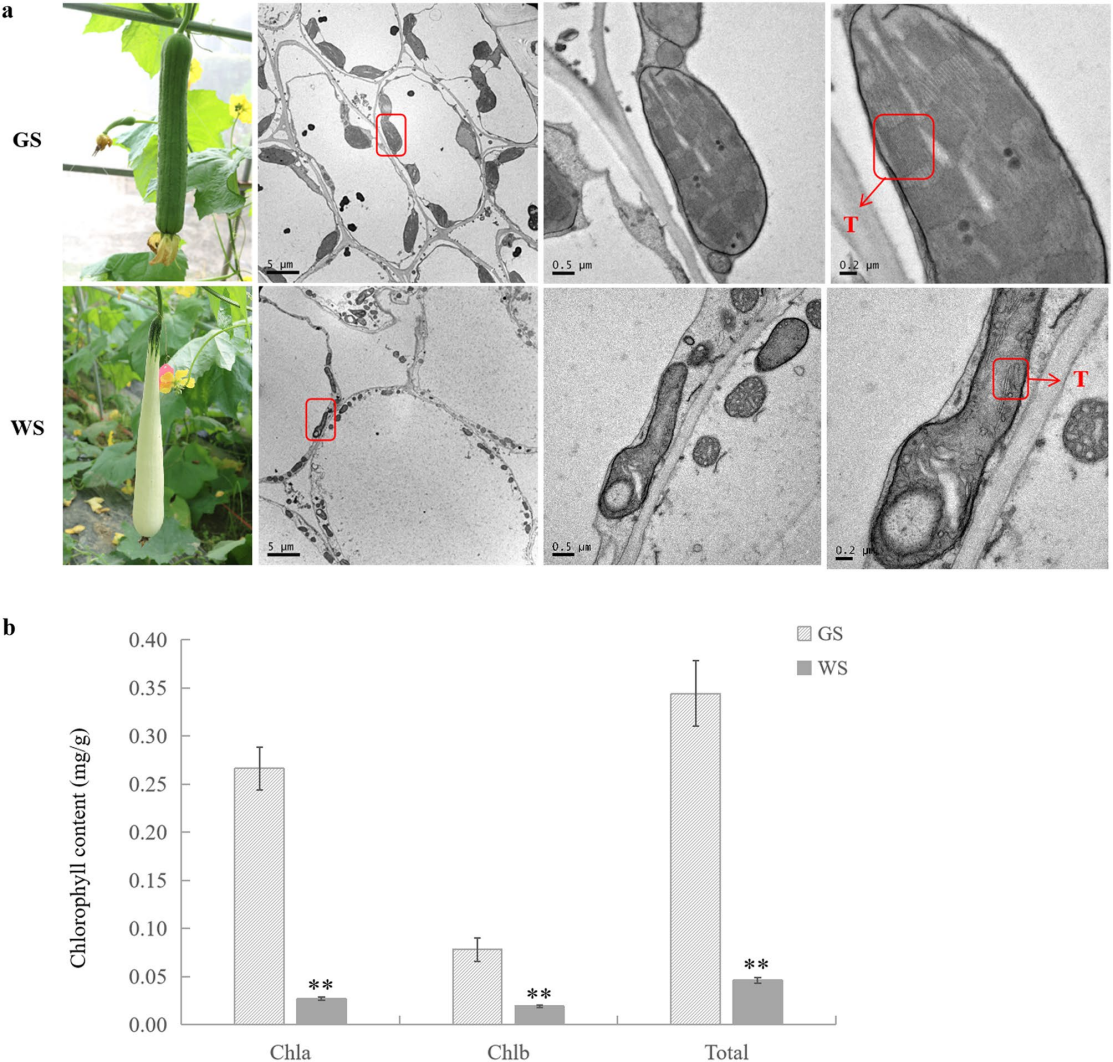
Phenotypic characterization, chloroplast ultrastructure and chlorophyll contents of WS and GS used in this study. (**a**) Phenotypic characterization and chloroplast ultrastructure of WS and GS. “T” indicates thylakoid. Less number of chloroplast and thylakoid were observed in WS than in GS. (**b**) Chlorophyll contents of WS and GS. The contents of Chla, Chlb and total Chl in WS were significantly reduced in WS compared with GS. “**” indicates chlorophyll contents are significantly different between WS and GS at *p* < 0.01 by T-test using SAS 8.0 software.

Mature fruits of white skin (WS) are presenting white fruit skin color, with green color at the fruit base, and mature fruits of green skin (GS) are presenting green fruit skin color (Fig. 1a). A transmission electron microscopy analysis revealed there were fewer chloroplasts, with fewer thylakoids per chloroplast, in fruit skin cells of WS than in GS (Fig. 1a). The chlorophyll contents of fruit skin differed significantly between GS and WS. Specifically, the chlorophyll *a*, chlorophyll *b*, and total chlorophyll contents were respectively 0.266, 0.078, and 0.344 mg/g for GS, but were 0.0268, 0.0191, and 0.0459 mg/g for WS (Fig. 1b).

### Overview of RNA-Seq results

A total of 120.81 M raw reads (18.11 Gb raw data) were obtained for the GS fruit skin libraries, whereas 140.07 M raw reads (21.01 Gb raw data) were obtained for the WS fruit skin libraries. After filtering the data, 112.87 M valid reads (16.92 Gb valid data) and 130.00 M valid reads (19.51 Gb valid data) were retained for the GS and WS fruit skin libraries, respectively. Additionally, 106.15 M valid reads, accounting for 94.04% of the total valid reads in the GS fruit skin libraries, and 117.52 M valid reads, accounting for 90.40% of the total valid reads in the WS fruit skin libraries, were mapped to the *L. cylindrica* genome. The Q30 values exceeded 97.00% and the GC contents were 45.00%–46.00% for all libraries (Table 1). These results indicated that the RNA-Seq data were valid and appropriate for the subsequent analysis.

**Table 1:** Summary of the RNA-Seq data for the fruit skins of GS and WS.

Term	GS_1	GS_2	GS_3	GS_Total	WS_1	WS_2	WS_3	WS_Total
Raw reads	51,294,344	32,627,566	36,893,884	120,815,794	50,842,746	47,230,626	42,003,120	140,076,492
Raw base (Gb)	7.69	4.89	5.53	18.11	7.63	7.08	6.30	21.01
Valid reads	47,955,496	30,491,348	34,426,084	112,872,928	47,175,422	43,523,318	39,303,214	130,001,954
Valid base (Gb)	7.19	4.57	5.16	16.92	7.08	6.53	5.90	19.51
Valid reads ratio (%)	93.49	93.45	93.31	93.42	92.79	92.15	93.57	92.81
Mapped reads	44,237,418 (92.25%)	29,140,615 (95.57%)	32,776,290 (95.21%)	106,154,323 (94.04%)	44,016,939 (93.30%)	36,395,054 (83.62%)	37,108,385 (94.42%)	117,520,378 (90.40%)
Unique mapped reads	37,428,776 (78.05%)	25,029,482 (82.09%)	28,066,795 (81.53%)	90,525,053 (80.20%)	37,177,560 (78.81%)	30,533,533 (70.15%)	31,435,025 (79.98%)	99,146,118 (76.26%)
Q20 (%)	99.98	99.98	99.98	99.98	99.99	99.99	99.98	99.99
Q30 (%)	97.87	97.63	97.74	97.74	97.82	97.75	97.68	97.75
GC content (%)	46.00	45.50	45.00	45.50	45.00	46.00	45.00	45.33

### Identification and functional annotation of DEGs

**Figure 2:**
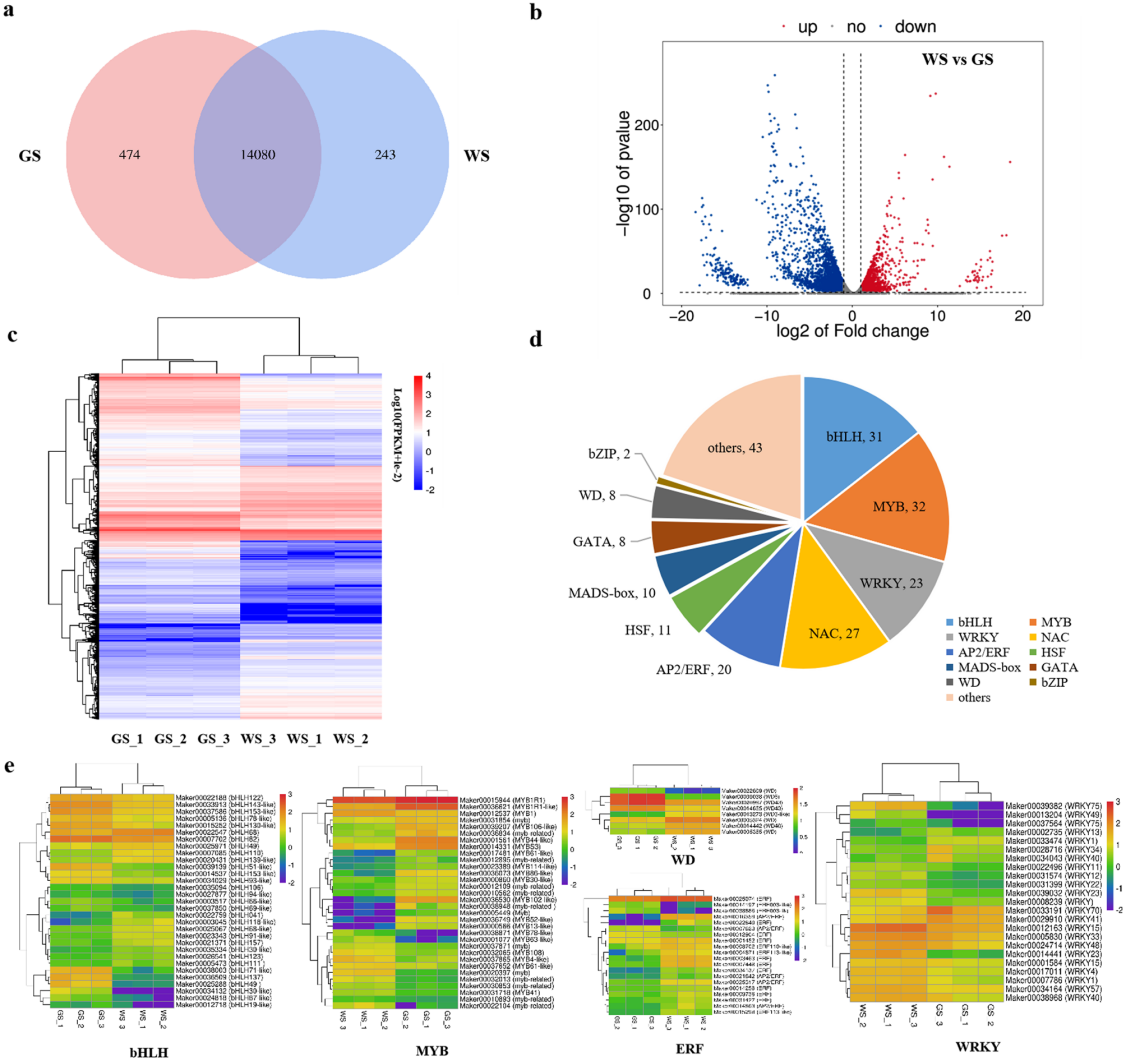
Analysis of mRNA-Seq results. (**a**) Venn diagram of genes detected as expressed at least one library. (**b**) Volcano map of 4,331 DEGs for WS and GS. (**c**) Heatmap of 4,331 DEGs for WS and GS. The heatmap of these DEGs were indicated by log_10_(FPKM + le^−2^) using OmicStudio tools at https://www.omicstudio.cn/tool. (**d**) Pie chart of 215 DEGs encoding transcription factors for WS and GS. (**e**) Heatmap of DEGs encoding TFs of bHLH, MYB, WD, ERF and WRKY for WS and GS. The heatmaps of these TFs were indicated by log_10_(FPKM + le^−2^) using OmicStudio tools at https://www.omicstudio.cn/tool.

In total, 14,797 genes were detected as expressed in at least one library (Fig. 2a and Table S3), of which 14,080 were expressed in both GS and WS fruit skins, whereas 474 and 243 genes were specifically expressed in GS and WS fruit skins, respectively (Fig. 2a). On the basis of |log_2_(fold-change)|> 1 and *p* < 0.05, 4331 genes that were differentially expressed between GS and WS fruit skins were identified, including 2442 down-regulated and 1889 up-regulated genes in WS fruit skins (relative to the expression levels in GS fruit skins) (Table S3). The volcano map and heatmap for these DEGs are presented in Fig. 2b,c.

The DEGs included 215 transcription factor genes, of which 132 and 83 were down-regulated and up-regulated, respectively, in WS fruit skins (relative to the expression levels in GS fruit skins) (Table S4). These transcription factor genes were revealed to belong to the *myeloblastosis* (*MYB*) (32), *basic/helix-loop-helix* (*bHLH*) (31), *NAM, ATAF and CUC* (*NAC*) (27), *WRKY* (23), *APETALA2/ethylene response factor* (*AP2*/*ERF*) (20), *heat shock transcription factor* (*HSF*) (11), *MCM1/AGAMOUS/DEFICIENS/SRF*(*MADS*)*-box* (10), *GATA* (8), *WD* (8), *basic leucine zipper* (*bZIP*) (2), and other (43) families (Table S4 and Fig. 2d). The expression level of *bHLH*, *MYB*, *WD*, *ERF*, and *WRKY* transcription factor genes were shown in Fig. 2e.

**Figure 3:**
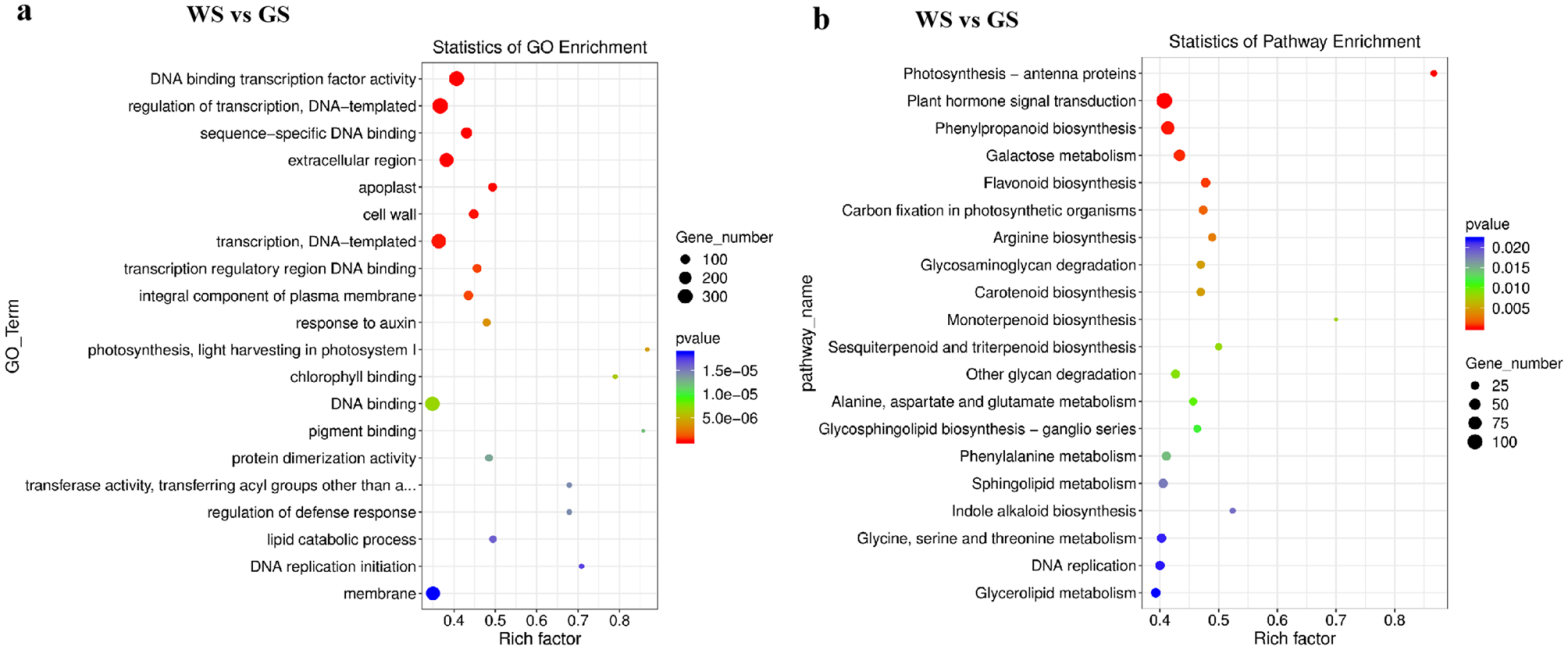
GO and KEGG pathway enrichment analysis of DEGs for WS and GS. (**a**) Top 20 GO terms enriched for DEGs. (**b**) Top 20 KEGG pathways enriched for DEGs. The size of each circle represents the number of significantly DEGs enriched in the corresponding GO term and pathway. The rich factor was calculated using the number of enriched genes divided by the total number of background genes in the corresponding GO term and pathway.

The DEGs were functionally characterized following gene ontology (GO) and Kyoto Encyclopedia of Genes and Genomes (KEGG) enrichment analyses. The significantly enriched GO terms were DNA binding transcription factor activity (GO:0003700), sequence-specific DNA binding (GO:0043565), extracellular region (GO:0005576), apoplast (GO:0048046), cell wall (GO:0005618), transcription regulatory region DNA binding (GO:0044212), integral component of plasma membrane (GO:0005887), response to auxin (GO:0009733), photosynthesis, light harvesting in photosystem I (GO:0009768), chlorophyll binding (GO:0016168), DNA binding (GO:0003677), and pigment binding (GO:0031409) (Fig. 3a). The significantly enriched KEGG pathways were photosynthesis-antenna proteins (ko00196), plant hormone signal transduction (ko04075), phenylpropanoid biosynthesis (ko00940), galactose metabolism (ko00052), flavonoid biosynthesis (ko00941), carbon fixation in photosynthetic organisms (ko00710), arginine biosynthesis (ko00220), glycosaminoglycan degradation (ko00531), carotenoid biosynthesis (ko00906), monoterpenoid biosynthesis (ko00902), and sesquiterpenoid and triterpenoid biosynthesis (ko00909) (Fig. 3b).

### DEGs involved in chlorophyll metabolism

**Figure 4:**
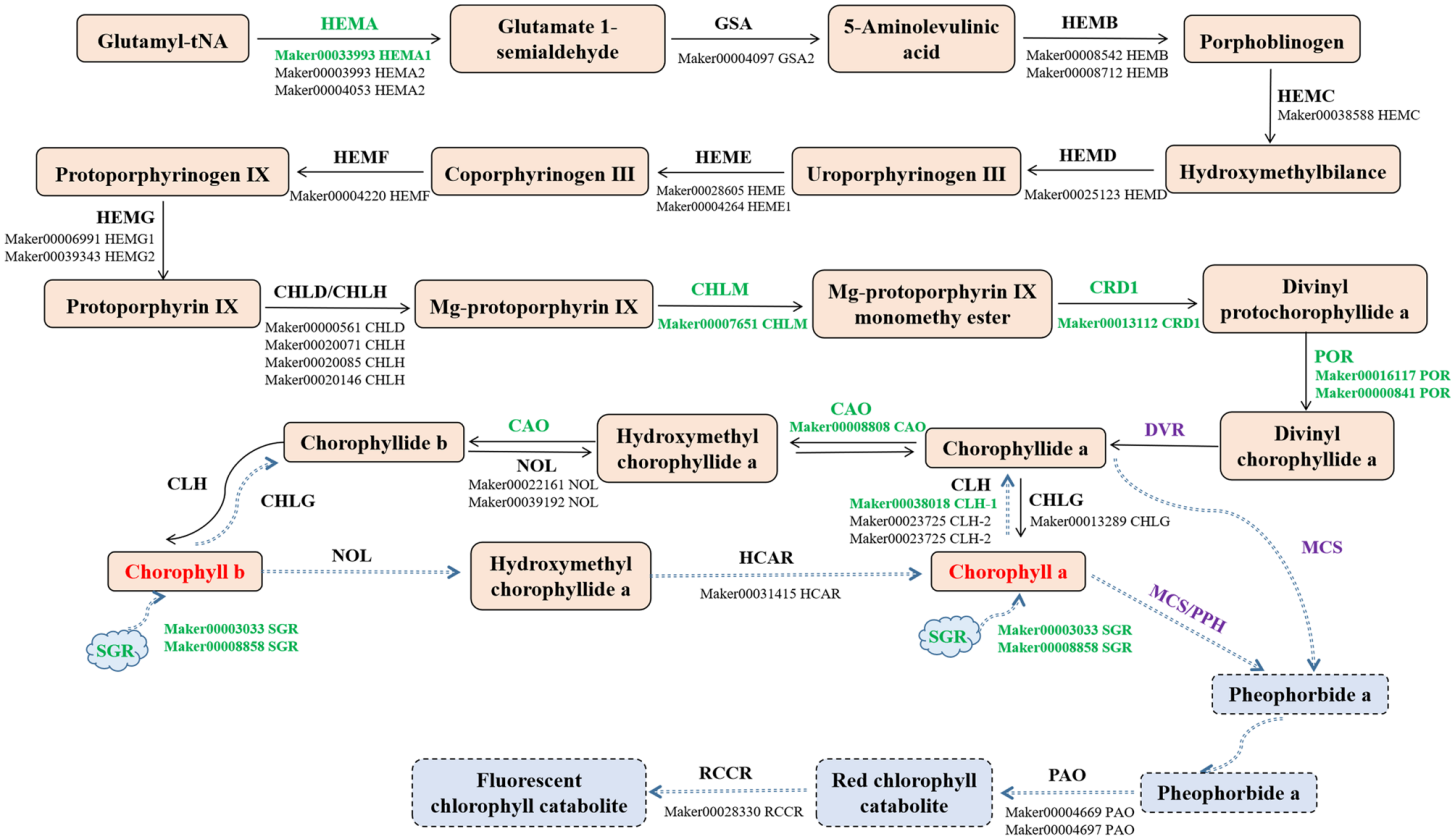
DEGs involved in chlorophyll metabolism. This pathway was constructed according to the previous reports of chlorophyll biosynthesis and degradation in higher plants. *HEMA* glutamyl-tRNA reductase, *GSA* glutamate-1-semialdehyde 2,1-aminomutase, *HEMB* delta-aminolevulinic acid dehydratase, *HEMC* porphobilinogen deaminase, *HEMD* uroporphyrinogen-III synthase, *HEME* uroporphyrinogen decarboxylase, *HEMF* oxygen-dependent coproporphyrinogen-III oxidase, *HEMG* protoporphyrinogen oxidase, *CHLD* magnesium-chelatase subunit ChlD, *CHLH* magnesium-chelatase subunit ChlH, *CHLM* magnesium protoporphyrin IX methyltransferase, *CRD1* magnesium-protoporphyrin IX monomethyl ester [oxidative] cyclase, *POR* protochlorophyllide reductase, *CHLG* chlorophyll synthase, *CAO* chlorophyllide a oxygenase, *NOL* chlorophyll(ide) b reductase, *CLH* chlorophyllase, *HCAR* 7-hydroxymethyl chlorophyll a reductase, *SGR* protein STAY-GREEN, *RCCR* red chlorophyll catabolite reductase.

The expression levels of genes involved in chlorophyll metabolism were analyzed (Fig. 4). The expression levels of chlorophyll biosynthesis-related genes, including *HEMA* (glutamyl-tRNA reductase 1, Maker00033993), *CHLM* (magnesium protoporphyrin IX methyltransferase, Maker00007651), *CRD1* (magnesium protoporphyrin IX monomethyl [oxidative] ester cyclase, Maker00013112), *POR* (protochlorophyllide reductase, Maker00016117 and Maker00000841), and *CAO* (chlorophyllide *a* oxygenase, Maker00008808), were significantly down-regulated in WS (Fig. 4 and Table 2). Additionally, the expression levels of genes contributing to chlorophyll degradation, such as *CLH1* (chlorophyllase-1-like, Maker00038018) and two *SGR* genes (protein STAY-GREEN, Maker00003033 and Maker00008858), were also down-regulated in WS (Fig. 4 and Table 2). The fact that the expression of all these genes involved in chlorophyll metabolism was down-regulated in WS implied chlorophyll biosynthesis was repressed in the fruit skins of WS sponge gourd.

**Table 2:** Differentially expressed genes involved in chlorophyll metabolism or chloroplast development and protection.

Function	Gene ID	Gene annotation	Log_2_ (FC)	Up/down-regulation
Chlorophyll metabolism	Maker00033993	Glutamyl-tRNA reductase 1	− 1.72	Down
	Maker00007651	Magnesium protoporphyrin IX methyltransferase	− 2.29	Down
	Maker00013112	Magnesium-protoporphyrin IX monomethyl ester [oxidative] cyclase	− 3.52	Down
	Maker00016117	Protochlorophyllide reductase	− 3.10	Down
	Maker00000841	Protochlorophyllide reductase-like	− 2.53	Down
	Maker00038018	Chlorophyllase-1-like	− 6.92	Down
	Maker00008808	Chlorophyllide a oxygenase	− 2.46	Down
	Maker00003033	Protein STAY-GREEN	− 4.51	Down
	Maker00008858	Protein STAY-GREEN LIKE	− 2.61	Down
Chlorophyll a/b binding protein	Maker00004449	Chlorophyll a-b binding protein CP26	− 4.43	Down
	Maker00006219	Chlorophyll a-b binding protein 151	− 4.06	Down
	Maker00008406	Chlorophyll a-b binding protein CP24 10A	− 4.18	Down
	Maker00011659	Chlorophyll a-b binding protein of LHCII type 1-like	− 5.04	Down
	Maker00012823	Chlorophyll a-b binding protein CP29.1	− 3.20	Down
	Maker00022071	Chlorophyll a-b binding protein 8	− 4.82	Down
	Maker00022517	Chlorophyll a-b binding protein P4	− 4.07	Down
	Maker00022767	Chlorophyll a/b-binding protein 6	− 3.92	Down
	Maker00036888	Chlorophyll a-b binding protein P4	− 6.50	Down
	Maker00039757	Chlorophyll a-b binding protein 6A	− 4.79	Down
Photosynthesis	Maker00013010	Oxygen-evolving enhancer protein 1	− 1.61	Down
	Maker00004248	Oxygen-evolving enhancer protein 2	− 1.74	Down
	Maker00017083	Photosystem I reaction center subunit IV	− 6.42	Down
	Maker00012955	Photosystem I reaction center subunit N	− 2.17	Down
	Maker00012025	Photosystem I reaction center subunit psaK	− 2.56	Down
	Maker00017196	Photosystem I reaction center subunit XI	− 3.76	Down
	Maker00019563	Photosystem I subunit O	− 4.17	Down
	Maker00014866	Photosystem II reaction center W protein	− 2.74	Down
	Maker00005035	Photosystem II stability/assembly factor HCF136	1.22	Up
	Maker00013798	Photosystem II stability/assembly factor HCF136	− 1.24	Down
	Maker00025426	psbP domain-containing protein 3	− 1.50	Down
	Maker00032710	psbP domain-containing protein 6	− 1.47	Down
Chloroplast development	Maker00013097	BEL1-like homeodomain protein 4	− 2.74	Down
	Maker00009063	BEL1-like homeodomain protein 4	− 2.88	Down
	Maker00005387	BEL1-like homeodomain protein 1	− 1.08	Down
	Maker00014487	BEL1-like homeodomain protein 7	1.12	Up
	Maker00000735	BEL1-like homeodomain protein 1	− 2.04	Down
	Maker00016760	Homeobox protein knotted-1-like 3 isoform X2	− 1.88	Down
	Maker00034080	Homeobox protein knotted-1-like 3 isoform X1	− 1.56	Down
	Maker00025590	Homeobox protein knotted-1-like 6	− 2.02	Down
	Maker00003022	Homeobox protein knotted-1-like 7	− 1.32	Down
	Maker00025662	Homeobox protein knotted-1-like 6	− 1.37	Down
	Maker00016354	Homeobox protein knotted-1-like 2	− 13.47	Down
	Maker00008553	Two-component response regulator ARR17-like isoform X1	− 4.24	Down
	Maker00017452	Auxin response factor 19-like	− 1.15	Down
	Maker00001191	Auxin response factor 4	3.68	Up
	Maker00008400	Auxin response factor 8 protein	2.47	Up
	Maker00035790	Auxin response factor 6-like	1.63	Up
	Maker00011775	Auxin response factor 3 isoform X2	1.31	Up
Chloroplast protection (peroxidase)	Maker00001025	Peroxidase 2-like	− 9.68	Down
	Maker00029979	Peroxidase 72-like	− 9.19	Down
	Maker00000296	Peroxidase 4-like	− 5.91	Down
	Maker00001433	Peroxidase 2-like	− 4.59	Down
	Maker00039543	Peroxidase 55-like	− 7.35	Down
	Maker00013160	Peroxidase 64-like	− 4.60	Down
	Maker00033633	Peroxidase 64	− 7.21	Down
	Maker00036048	Peroxidase 5	− 2.36	Down
	Maker00033470	Peroxidase 21-like	− 6.21	Down
	Maker00013033	Peroxidase 73-like	− 5.32	Down
	Maker00000305	Peroxidase 2-like	− 4.21	Down
	Maker00000884	Peroxidase 2-like	− 3.12	Down
	Maker00000788	Peroxidase 3-like	− 2.27	Down
	Maker00007750	Peroxidase P7-like	− 3.50	Down
	Maker00010179	Peroxidase 11-like isoform X1	− 4.38	Down
	Maker00006240	Peroxidase 3	− 3.45	Down
	Maker00006682	Peroxidase 47	− 1.30	Down
	Maker00023047	Peroxidase 4	− 1.25	Down
	Maker00001246	Peroxidase 2	− 2.70	Down
	Maker00001025	Peroxidase 2-like	− 9.68	Down
	Maker00029979	Peroxidase 72-like	− 9.19	Down
	Maker00025909	Peroxidase 11 isoform X1	1.98	Up
	Maker00039275	Peroxidase 55	6.16	Up
	Maker00036599	Peroxidase 19	1.83	Up
	Maker00006262	Peroxidase 6	1.67	Up

### DEGs involved in chlorophyll binding and photosynthesis

The RNA-Seq results revealed the down-regulated expression of 10 genes in WS fruit skins (relative to the expression levels in GS fruit skins) encoding the chlorophyll *a*/*b*-binding protein (CAB) (Maker00004449, Maker00006219, Maker00008406, Maker00011659, Maker00012823, Maker00022071, Maker00022517, Maker00022767, Maker00036888, and Maker00039757), with log_2_(fold-change) values ranging from − 3.20 to − 6.50 (Table 2).

Twelve DEGs related to photosynthesis were detected (Table 2). With the exception of the up-regulated expression of Maker00005035 (photosystem II stability/assembly factor), the expression levels of other genes, including two genes (Maker00013010 and Maker00004248) encoding the oxygen-evolving enhancer protein, five genes (Maker00017083, Maker00012955, Maker00012025, Maker00017196, and Maker00019563) encoding the photosystem I reaction center subunit, one gene (Maker00014866) encoding the photosystem II reaction center W protein, one gene (Maker00013798) encoding the photosystem II stability/assembly factor, and two genes (Maker00025426 and Maker00032710) encoding the psbP domain-containing protein, were down-regulated in WS fruit skins (relative to the expression levels in GS fruit skins). These results indicated that photosynthetic activities were substantially lower in fruit skins of WS than in GS.

### DEGs involved in chloroplast development and protection

Chlorophyll biosynthesis is affected by processes related to chloroplast development and protection. Several genes that control chloroplast development, including *BEL1-LIKE HOMEODOMAIN*, *KNAT*, *ARR-like*, and *ARF* genes, have been thoroughly investigated. In this study, five *BEL1-LIKE HOMEODOMAIN* genes (Maker00013097, Maker00009063, Maker00005387, Maker00014487, and Maker00000735) were among the identified DEGs. In WS fruit skins, the Maker00014487 expression level was up-regulated, whereas the remaining four *BEL1-LIKE HOMEODOMAIN* genes had down-regulated expression levels. The expression levels of six genes (Maker00016760, Maker00034080, Maker00025590, Maker00003022, Maker00025662, and Maker00016354) encoding the homeobox protein knotted-1-like (KNAT) and one gene (Maker00008553) encoding ARR17-like were down-regulated in WS fruit skins. Additionally, five ARF-encoding genes were identified, including four (Maker00001191, Maker00008400, Maker00035790, and Maker00011775) up-regulated genes and one (Maker00017452) down-regulated gene in WS fruit skins (Table 2). Chloroplast protection is largely associated with peroxidase gene expression levels. In this study, 25 peroxidase genes were differentially expressed between the WS and GS fruit skins, of which the expression levels of 21 and 4 genes were respectively down-regulated and up-regulated in WS fruit skins (relative to the expression levels in GS fruit skins). These results indicated that the expression of genes involved in chloroplast development and protection was mostly repressed in the WS fruit skins, thereby limiting chlorophyll biosynthesis.

### DEGs involved in flavonoid biosynthesis

Twenty DEGs (18 down-regulated and 2 up-regulated) associated with flavonoid biosynthesis were detected in this study (Table 3). These DEGs encode phenylalanine ammonia-lyase (PAL), trans-cinnamate 4-monooxygenase (C4H), 4-coumarate-CoA ligase (4CL), chalcone synthase (CHS), chalcone-flavonone isomerase (CHI), flavanone 3-hydroxylase (F3H), flavonoid 3′-monooxygenase (F3′H), flavonoid 3′,5′-methyltransferase (F3′5′H), dihydroflavonol-4-reductase (DFR), anthocyanidin 3-O-glucosyltransferase (UFGT), and malonyl-CoA:anthocyanidin 5-O-glucoside-6″-O-malonyltransferase (5MAT). Compared with the corresponding expression levels in GS fruit skins, Maker00032587 (4CL7) and Maker00013821 (F3H) expression levels were up-regulated, whereas the remaining 18 DEGs had down-regulated expression levels in WS fruit skins. These results indicated flavonoid biosynthesis was largely restricted in the WS sponge gourd fruit skins.

**Table 3:** Differentially expressed genes involved in flavonoid biosynthesis.

Gene ID	Gene annotation	Log_2_ (FC)	Up/down-regulation
Maker00009938	Phenylalanine ammonia-lyase 5	− 4.99	Down
Maker00039849	Phenylalanine ammonia-lyase G4	− 16.37	Down
Maker00012461	Phenylalanine ammonia-lyase	− 4.19	Down
Maker00001230	Trans-cinnamate 4-monooxygenase	− 2.07	Down
Maker00036252	4-coumarate-CoA ligase	− 1.31	Down
Maker00038653	4-coumarate-CoA ligase 1-like	− 5.11	Down
Maker00014265	4-coumarate-CoA ligase 2-like	− 3.04	Down
Maker00001858	4-coumarate-CoA ligase 2	− 5.82	Down
Maker00029489	4-coumarate-CoA ligase 2	− 1.16	Down
Maker00029496	4-coumarate-CoA ligase 2	− 1.96	Down
Maker00032587	4-coumarate-CoA ligase-like 7	1.12	Up
Maker00001799	Chalcone synthase 2	− 5.88	Down
Maker00028599	Chalcone–flavonone isomerase 3 isoform X1	− 2.13	Down
Maker00013821	Flavonol synthase/flavanone 3-hydroxylase-like	1.22	Up
Maker00021976	Flavonoid 3'-monooxygenase	− 14.49	Down
Maker00017833	Flavonoid 3',5'-methyltransferase-like	− 5.23	Down
Maker00017687	Flavonoid 3',5'-methyltransferase-like	− 5.00	Down
Maker00026546	Dihydroflavonol-4-reductase	− 1.10	Down
Maker00029804	Anthocyanidin 3-O-glucosyltransferase 5-like	− 4.59	Down
Maker00001398	Malonyl-CoA: anthocyanidin 5-O-glucoside-6''-O-malonyltransferase-like	− 3.10	Down

### Overview of small RNA (sRNA) sequencing data

A total of 34,731,219 and 34,986,225 raw reads, including 15,290,221 and 16,301,179 unique reads, were generated through the sRNA high-throughput sequencing of the WS and GS fruit skin libraries, respectively. After removing low-quality reads and adapter contaminants, 24,406,680 and 26,121,851 valid reads, including 13,512,549 and 14,453,503 unique reads, were retained for the WS and GS fruit skin libraries, respectively (Table 4).

**Table 4:** Summary of the small RNA sequencing results for the fruit skins of GS and WS.

Sample	Term	Raw reads	3ADT and length filter	Junk reads	Rfam	mRNA	Repeats	Valid reads
WS_1	Total	11,049,078	1,799,596	42,566	346,573	1,498,848	2,646	7,578,791
	% of total	100.00	16.29	0.39	3.14	13.57	0.02	68.59
	Unique	4,900,499	513,380	31,381	5,768	68,613	117	4,283,785
	% of unique	100.00	10.48	0.64	0.12	1.40	0.00	87.42
WS_2	Total	9,340,471	1,635,089	35,643	284,353	1,235,856	2,152	6,317,995
	% of total	100.00	17.51	0.38	3.04	13.23	0.02	67.64
	Unique	4,141,723	425,368	26,423	4,733	57,868	103	3,629,336
	% of unique	100.00	10.27	0.64	0.11	1.40	0.00	87.63
WS_3	Total	14,341,670	1,307,252	69,814	495,752	2,236,550	3,068	10,509,894
	% of total	100.00	9.12	0.49	3.46	15.59	0.02	73.28
	Unique	6,247,999	497,531	47,451	8,674	98,172	171	5,599,428
	% of unique	100.00	7.96	0.76	0.14	1.57	0.00	89.62
GS_1	Total	12,480,603	1,907,130	56,754	518,118	1,648,373	5,770	8,686,544
	% of total	100.00	15.28	0.45	4.15	13.21	0.05	69.60
	Unique	5,359,252	576,844	39,385	8,726	67,444	175	4,670,363
	% of unique	100.00	10.76	0.73	0.16	1.26	0.00	87.15
GS_2	Total	9,327,741	800,777	56,796	240,815	1,015,434	1,623	7,360,534
	% of Total	100.00	8.58	0.61	2.58	10.89	0.02	78.91
	Unique	4,831,166	353,209	41,983	4,932	52,200	80	4,380,774
	% of unique	100.00	7.31	0.87	0.10	1.08	0.00	90.68
GS_3	Total	13,177,881	1,215,300	80,098	389,866	1,646,244	3,239	10,074,773
	% of total	100.00	9.22	0.61	2.96	12.49	0.02	76.45
	Unique	6,110,761	572,590	54,612	6,728	77,121	133	5,402,366
	% of unique	100.00	9.37	0.89	0.11	1.26	0.00	88.41

**Figure 5:**
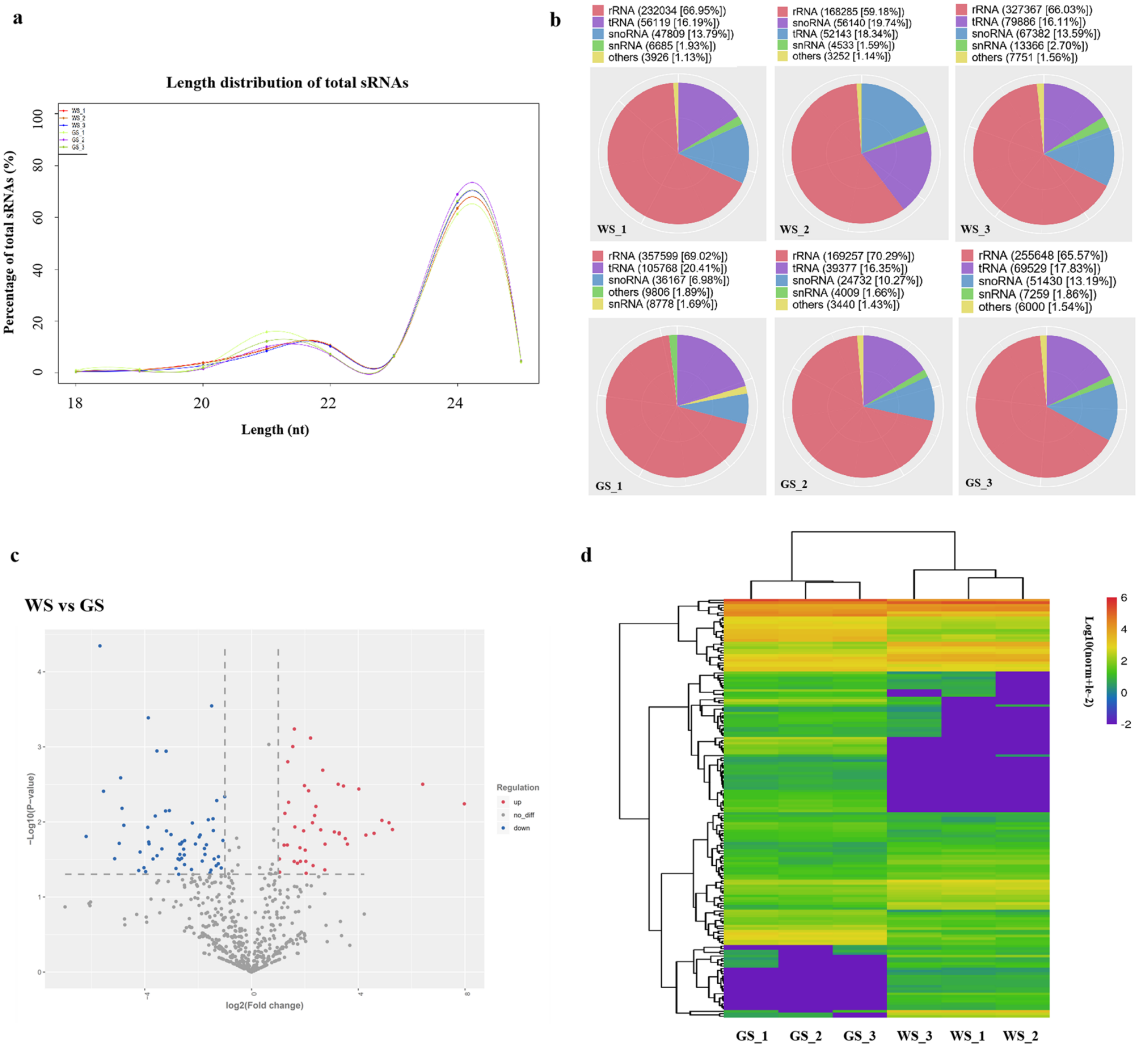
Analysis of small RNA sequencing results. (**a**) Length distribution of small RNA in 18–25 nt in WS and GS libraries. The 24 nt sRNA in length was the most abundant in all libraries of WS and GS. (**b**) Classification of non-coding RNAs by blasting to Rfam database. Five classification of non-coding RNAs were observed, with rRNA accounting for the majority, followed by tRNA, snoRNA, snRNA, and others. (**c**) Volcano map of 167 DE-miRNAs for WS and GS. (**d**) Heatmap of 167 DE-miRNAs for WS and GS. The heatmap of these DE-miRNAs was indicated by log_10_(Norm + le^−2^) using OmicStudio tools at https://www.omicstudio.cn/tool.

The length distribution of the sRNA sequences (18–25 nt) was analyzed (Fig. 5a). More than 85% of all sRNA sequences were 21–24 nt long in the GS and WS fruit skin libraries, with 24-nt being the most common length (Fig. 5a). The sRNA sequences were used as queries for a BLAST search of the Rfam database to obtain the non-coding RNAs (e.g., rRNAs, tRNAs, snoRNAs, snRNAs, and others). For all six libraries, rRNAs were the most abundant non-coding RNAs, followed by the tRNAs, snoRNAs, snRNAs, and others (Fig. 5b).

### Identification of miRNAs and analysis of their differential expression

A total of 862 miRNAs, with 826 pre-miRNA sequences, were predicted in the analyzed libraries (Table S5). Details regarding these miRNAs, including sequences, lengths, pre-miRNA locations and sequences, hairpin lengths, minimal free energy index (MFEI), and expression levels are listed in Table S5.

The differentially expressed miRNAs (DE-miRNAs) were identified according to the following criteria: |log_2_(fold-change)|> 1 and *p* < 0.05. A total of 167 DE-miRNAs were detected, with 70 up-regulated and 97 down-regulated miRNAs in WS fruit skins (relative to the expression levels in GS fruit skins) (Table S5). For example, five miR159 family members (cme-miR159a_1ss17CA, cme-miR159a_1ss8TC, cme-miR159a_R-1, cme-miR159a_R-1_1ss12GT, and cme-miR159a_R-1_1ss19CT), four miR172 family members (cme-miR172b, cme-miR172e, cme-MIR172e-p5, and mtr-miR172c-5p_1ss9AT), eight miR319 family members (ath-miR319a_1ss21TA, cme-miR319a, cme-miR319a_R + 2, cme-MIR319a-p3_1ss2TC, cme-MIR319b-p5, cme-miR319c_R + 2_1ss20TC, mdm-miR319c-5p_L + 1R-1_2ss7C-20GC, and mdm-miR319d_L-1R + 1_1ss21AC) had up-regulated expression levels in the WS fruit skin libraries. In contrast, nine miR166 family members (cme-miR166a, cme-miR166a_1ss20CT, cme-miR166a_2ss19CT21CG, cme-miR166a_L + 2R-2, aly-miR166a-3p_R + 2_1ss20CA, ath-miR166a-5p_1ss4CA, ath-miR166a-5p_2ss4CA10TG, ath-miR166a-5p_R + 2_2ss4CA10 TG, and mtr-miR166e-5p_R + 1), six miR171 family members (cme-MIR171b-p5, cme-miR171c, cme-miR171c_L + 1, cme-miR171c_L + 3, cme-MIR171d-p5, and csi-miR171c-5p), five miR390 family members (aly-miR390a-5p_R + 1_1ss16AG, ath-miR390a-3p_1ss8CT, ath-miR390b-3p_R + 1_1ss19CT, cme-miR390a, and cme-miR390a_R + 1), and two miR393 family members (cme-miR393a and cme-MIR393a-p3) had down-regulated expression levels in the WS fruit skin libraries. The volcano map and heatmap for these DE-miRNAs are provided in Fig. 5c,d.

### Prediction of miRNA target genes by degradome sequencing

In total, 4,042 target genes were predicted for 476 miRNAs on the basis of degradome sequencing data (Table S6). The number of miRNA target genes ranged from 1 (cme-MIR169e-p5_2ss22TC23AG) to 145 (PC-3p-10341_215). For example, three target genes (Maker00007474, Maker00014332, and Maker00021968) were detected for cme-miR159a, five target genes (Maker00016366, Maker00016802, Maker00025551, Maker00030959, and Maker00035519) were detected for cme-miR172e, and eight target genes (Maker00001359, Maker00001668, Maker00003319, Maker00004449, Maker00013716, Maker00013995, Maker00018145, and Maker00037385) were detected for cme-miR393a (Table S6).

Several miRNAs targeted a single gene. For example, Maker00012076 was identified as the target gene for aly-miR166a-3p_R + 2_1ss20CA, cme-miR166a_L + 2R-2, and mes-miR159a-5p_1ss8GC (Table S6). Moreover, aly-miR167d-3p_2ss9CT20GA targeted Maker00036436, with two splice sites (Maker00036436:1704 and Maker00036436:603) (Table S6).

### Network of miRNAs and their target genes regulating sponge gourd fruit skin coloration

**Figure 6:**
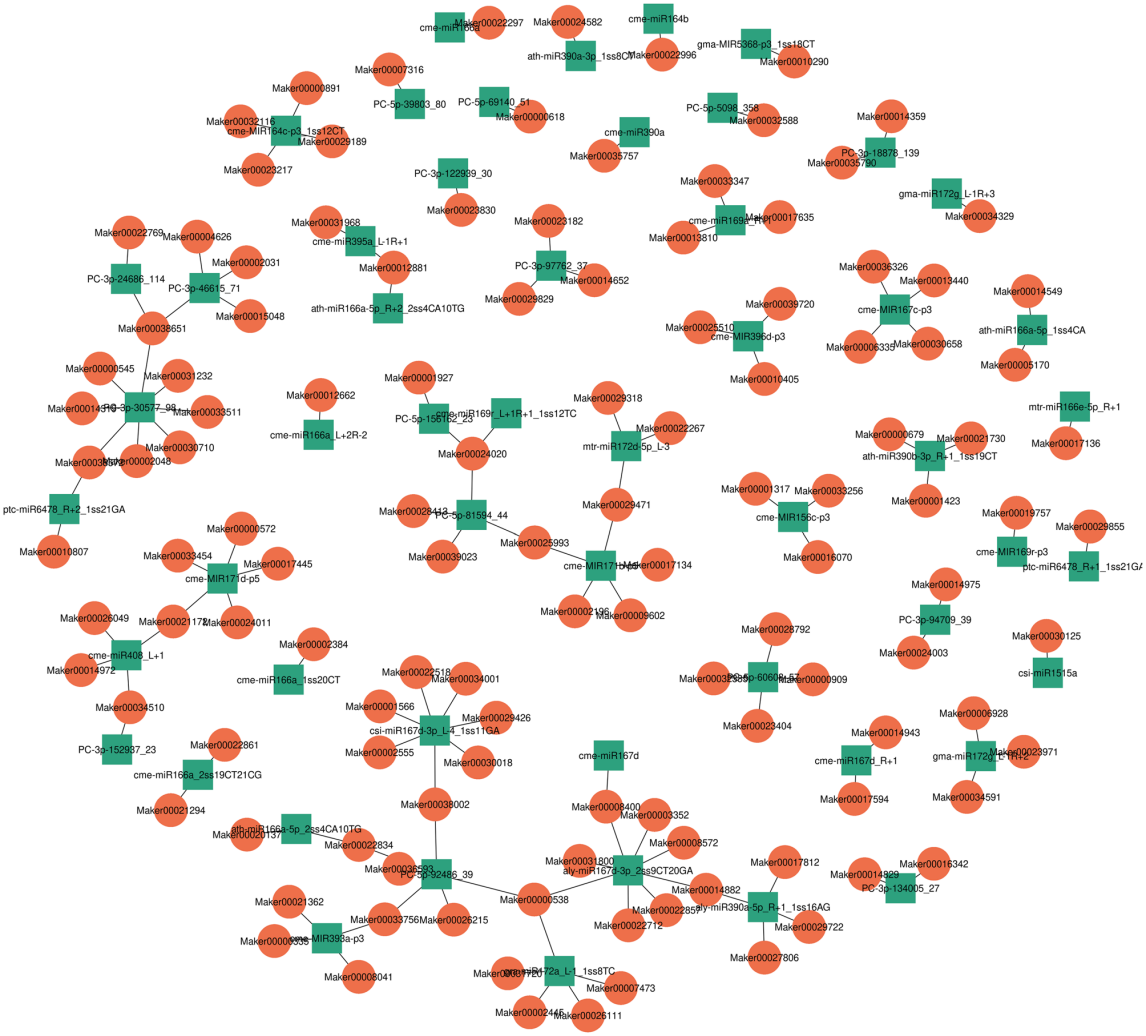
Network plot of 53 down-regulated miRNAs and their 133 up-regulated target genes. The miRNAs in green square indicates the down-regulated miRNAs in WS compared with GS and the genes in red circle indicates the up-regulated target genes in WS compared with GS. This network plot was drawn using Cytoscape.

**Figure 7:**
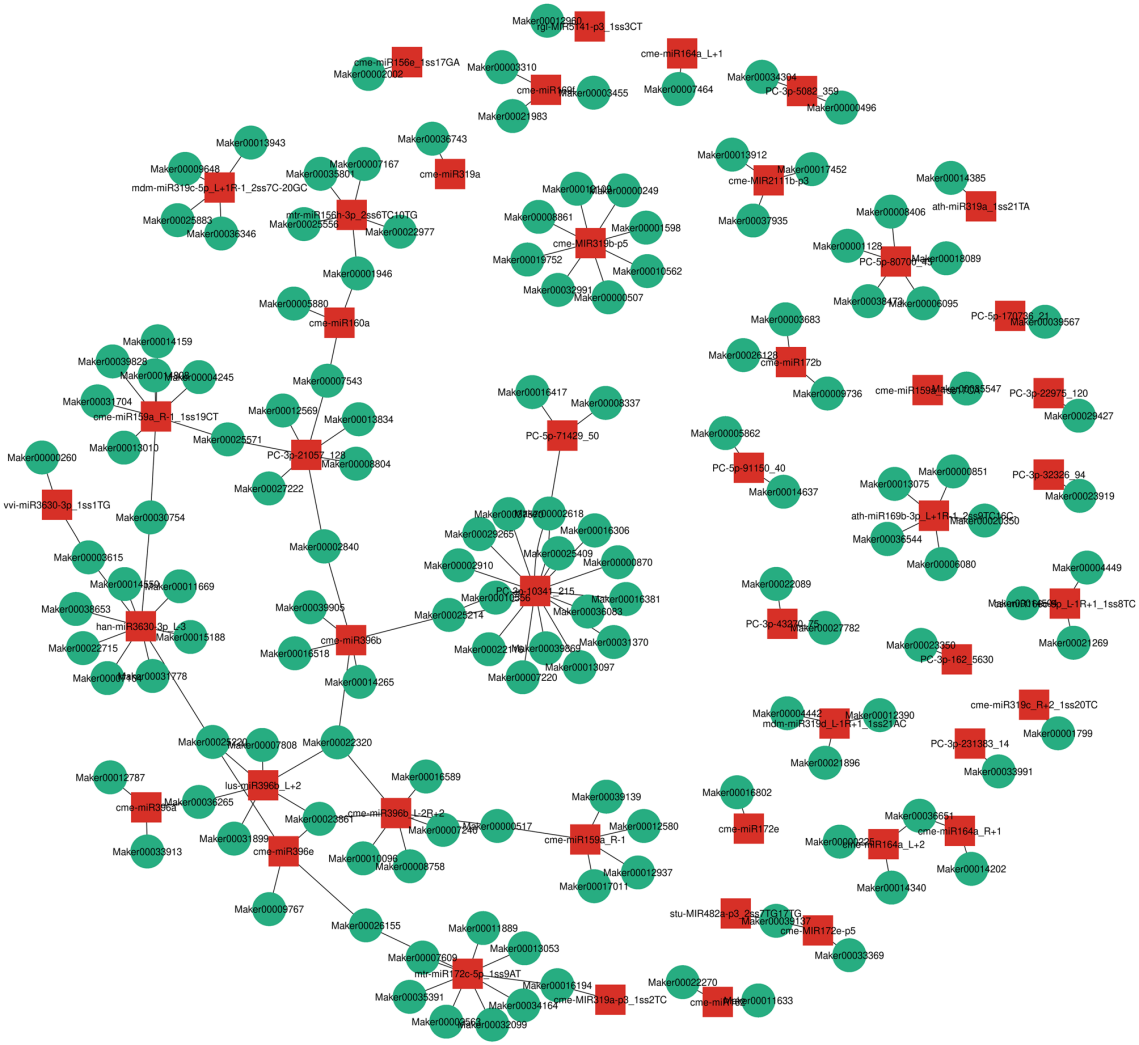
Network plot of 46 up-regulated miRNAs and their 141 down-regulated target genes. The miRNAs in red square indicates the up-regulated miRNAs in WS compared with GS and the genes in the green circle indicates the down-regulated target genes in WS compared with GS. This network plot was drawn using Cytoscape.

To reveal the regulatory roles of the DE-miRNAs and DEGs associated with sponge gourd fruit skin coloration, 125 DE-miRNAs and their 521 differentially expressed target genes were examined (Table S7). A network plot was constructed for the 53 down-regulated miRNAs and their 122 up-regulated target genes (Table S7 and Fig. 6). Another network plot was constructed for the 46 up-regulated miRNAs and their 141 down-regulated target genes (Table S7 and Fig. 7). The down-regulated cme-MIR156c-p3 targeted three up-regulated genes, Maker00001317 (mechanosensitive ion channel protein 6-like), Maker00016070 (homeobox-leucine zipper protein HAT4), and Maker00033256 (trihelix transcription factor PTL), whereas the down-regulated cme-MIR167c-p3 targeted four up-regulated genes, Maker00006335 (zinc finger protein CONSTANS-LIKE 9-like), Maker00013440 (serine acetyltransferase 2), Maker00030658 (indole-3-acetic acid-amido synthetase), and Maker00036326 (kinase-interacting family protein-like) (Table S5 and Fig. 6). Additionally, the up-regulated cme-miR159a_R-1 targeted five down-regulated genes, Maker00000517 (probable 2-carboxy-D-arabinitol-1-phosphatase), Maker00012580 (glutathione S-transferase), Maker00012937 (glutathione S-transferase), Maker00017011 (WRKY transcription factor 4-like protein), and Maker00039139 (transcription factor bHLH51-like), whereas the up-regulated csi-miR166c-5p_L-1R + 1_1ss8TC targeted three down-regulated genes, Maker00004449 (chlorophyll *a*/*b*-binding protein CP26), Maker00014504 (pheophytinase, chloroplastic), and Maker00021269 (inositol polyphosphate 5-phosphatase 2 isoform X1) (Table S7 and Fig. 7). These DE-miRNAs and their target genes provide valuable information useful for revealing the regulatory mechanism underlying sponge gourd fruit skin coloration.

### Network of DE-miRNAs and their differentially expressed target genes involved in chlorophyll metabolism and chloroplast development

In total, 22 DE-miRNAs and their 19 differentially expressed target genes related to chlorophyll metabolism and chloroplast development were identified (Table 5). For example, gra-miR172a_L-1_1ss8TC targeted Maker00016117, which encodes a protochlorophyllide reductase. Seven DE-miRNAs (csi-miR166c-5p_L-1R + 1_1ss8TC, cme-miR166a_L + 2R-2, aly-miR166a-3p_R + 2_1ss20CA, gma-miR172g_L-1R + 2, cme-miR393a, PC-3p-94709_39, and PC-5p-80700_45) targeted the gene encoding the chlorophyll *a*/*b*-binding protein. Additionally, cme-MIR156c-p3 targeted Maker00017196 (photosystem I reaction center subunit XI), PC-5p-81594_44 targeted Maker00014866 (photosystem II reaction center W protein), and cme-miR159a_R-1_1ss19CT targeted Maker00013010 (oxygen-evolving enhancer protein 1). Three DE-miRNAs, PC-3p-30577_98, ptc-miR6478_R + 2_2ss5CT21GA, and PC-3p-10341_215, respectively targeted Maker00034080, Maker00016760, and Maker00025409, all of which encode the homeobox protein knotted-1-like. Moreover, gma-MIR6300-p5_1ss20AG and PC-3p-10341_215 respectively targeted Maker00000735 and Maker00013097, both of which encode a BEL1-like homeodomain protein. Five DE-miRNAs (aly-miR167d-3p_2ss9CT20GA, cme-miR159a_1ss17CA, cme-miR167d, cme-MIR2111b-p3, and PC-3p-18878_139) targeted Maker00035790, Maker00008400, and Maker00017452, which encode ARF6, ARF8, and ARF19, respectively. Three DE-miRNAs, PC-3p-21057_128, gma-miR172g_L-1R + 2, and cme-MIR171d-p5, targeted three peroxidase genes (Maker00025909, Maker00036048, and Maker00013033). These results implied that MIR156, miR159, miR166, miR167, MIR171, miR172, and miR393 are crucial for chlorophyll metabolism and chloroplast development in sponge gourd.

**Table 5:** Differentially expressed miRNAs and their differentially expressed target genes associated with chlorophyll metabolism and chloroplast development.

miRNAs	Up/down for miRNAs	Target genes	Up/down for target genes	Target gene annotation
gra-miR172a_L-1_1ss8TC	Down	Maker00016117	Down	Protochlorophyllide reductase
PC-3p-94709_39	Down	Maker00006219	Down	Chlorophyll a-b binding protein 151
PC-5p-80700_45	Up	Maker00008406	Down	Chlorophyll a-b binding protein CP24 10A
cme-miR393a	Down	Maker00004449	Down	Chlorophyll a-b binding protein CP26
csi-miR166c-5p_L-1R + 1_1ss8TC	Up	Maker00004449	Down	Chlorophyll a-b binding protein CP26
gma-miR172g_L-1R + 2	Down	Maker00004449	Down	Chlorophyll a-b binding protein CP26
cme-miR166a_L + 2R-2	Down	Maker00012823	Down	Chlorophyll a-b binding protein CP29.1
aly-miR166a-3p_R + 2_1ss20CA	Down	Maker00012823	Down	Chlorophyll a-b binding protein CP29.1
cme-MIR156c-p3	Down	Maker00017196	Down	Photosystem I reaction center subunit XI
PC-5p-81594_44	Down	Maker00014866	Down	Photosystem II reaction center W protein
cme-miR159a_R-1_1ss19CT	Up	Maker00013010	Down	Oxygen-evolving enhancer protein 1
PC-3p-30577_98	Down	Maker00034080	Down	Homeobox protein knotted-1-like 3 isoform X1
ptc-miR6478_R + 2_2ss5CT21GA	Down	Maker00016760	Down	Homeobox protein knotted-1-like 3 isoform X2
PC-3p-10341_215	Up	Maker00025409	Down	Homeotic protein knotted-1-like isoform X2
gma-MIR6300-p5_1ss20AG	Down	Maker00000735	Down	BEL1-like homeodomain protein 1
PC-3p-10341_215	Up	Maker00013097	Down	BEL1-like homeodomain protein 4
aly-miR167d-3p_2ss9CT20GA	Down	Maker00008400	Up	Auxin response factor 8 protein
cme-miR159a_1ss17CA	Up	Maker00035790	Up	Auxin response factor 6-like
cme-miR167d	Down	Maker00008400	Up	Auxin response factor 8 protein
cme-MIR2111b-p3	Up	Maker00017452	Down	Auxin response factor 19-like
PC-3p-18878_139	Down	Maker00035790	Up	Auxin response factor 6-like
PC-3p-21057_128	Up	Maker00025909	Up	Peroxidase 11 isoform X1
gma-miR172g_L-1R + 2	Down	Maker00036048	Down	Peroxidase 5
cme-MIR171d-p5	Down	Maker00013033	Down	Peroxidase 73-like

### Network of DE-miRNAs and their differentially expressed target genes involved in flavonoid biosynthesis

Several DE-miRNAs and their differentially expressed target genes involved in flavonoid biosynthesis were identified (Table 6). For example, cme-MIR169r-p3, cme-miR396a, cme-miR159a_R-1, and PC-5p-39803_80 targeted four genes (Maker00015252, Maker00033913, Maker00039139, and Maker00034132) encoding bHLH transcription factors. Additionally, cme-MIR319b-p5 targeted two *MYB* genes (Maker00010562 and Maker00012109). Moreover, six miRNAs, cme-miR396b, han-miR3630-3p_L-3, ath-miR390b-3p_R + 1_1ss19CT, cme-miR166a_L + 2R-2, PC-3p-30577_98, and PC-5p-81594_44, targeted genes encoding 4CL (Maker00014265 and Maker00038653), which converts coumaroyl-CoA to p-coumaroyl-CoA. Furthermore, cme-miR319c_R + 2_1ss20TC targeted Maker00001799, which encodes CHS and converts p-coumaroyl-CoA to naringenin chalcone. All of these target genes were expressed at lower levels in WS than in GS fruit skins, which was indicative of less flavonoid biosynthesis in fruit skins of WS than in GS.

**Table 6:** Differentially expressed miRNAs and their differentially expressed target genes associated with flavonoid biosynthesis.

miRNAs	Up/down for miRNAs	Target genes	Up/down for target genes	Target gene annotation
cme-MIR169r-p3	Down	Maker00015252	Down	Transcription factor bHLH130-like isoform X2
cme-miR396a	Up	Maker00033913	Down	Transcription factor bHLH143-like isoform X1
cme-miR159a_R-1	Up	Maker00039139	Down	Transcription factor bHLH51-like
PC-5p-39803_80	Down	Maker00034132	Down	Transcription factor bHLH30-like
cme-MIR319b-p5	Up	Maker00010562	Down	myb-related protein 306-like
cme-MIR319b-p5	Up	Maker00012109	Down	myb-related protein 306-like
cme-miR319c_R + 2_1ss20TC	Up	Maker00001799	Down	Chalcone synthase 2
cme-miR396b	Up	Maker00014265	Down	4-Coumarate-CoA ligase 2-like
han-miR3630-3p_L-3	Up	Maker00038653	Down	4-Coumarate-CoA ligase 1-like
ath-miR390b-3p_R + 1_1ss19CT	Down	Maker00014265	Down	4-Coumarate-CoA ligase 2-like
cme-miR166a_L + 2R-2	Down	Maker00014265	Down	4-Coumarate-CoA ligase 2-like
PC-3p-30577_98	Down	Maker00014265	Down	4-Coumarate-CoA ligase 2-like
PC-5p-81594_44	Down	Maker00014265	Down	4-Coumarate-CoA ligase 2-like

### Quantitative real-time polymerase chain reaction (qRT-PCR) validation of the expression of genes associated with fruit color formation

**Figure 8:**
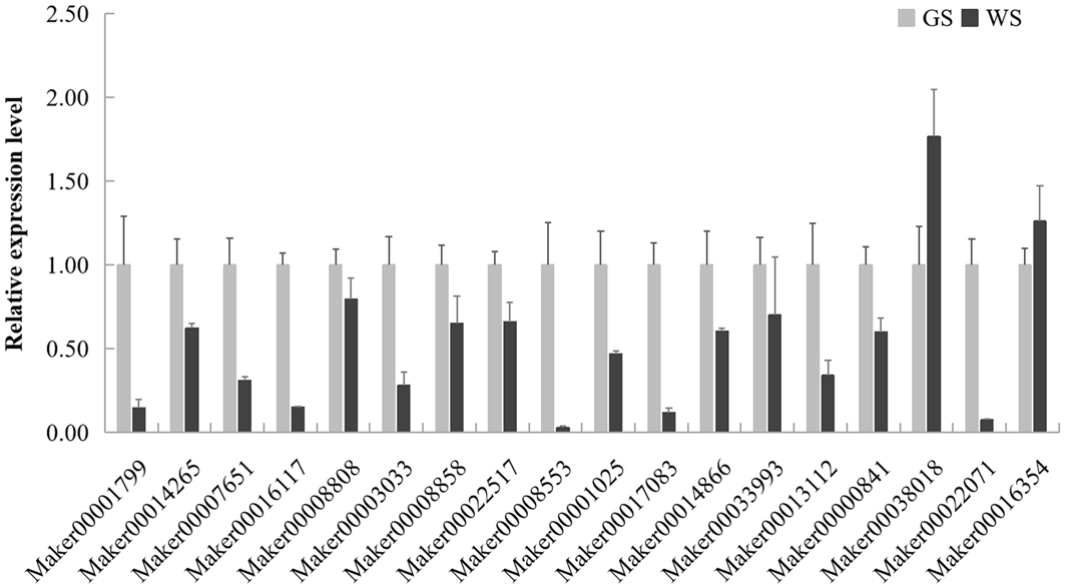
qRT-PCR validation of selected DEGs associated with fruit skin coloration. The relative expression level for each gene was the mean value of three biological replicates, and error bars means the standard error.

Eighteen DEGs were selected for a qRT-PCR validation of expression levels. Relative to the expression levels in GS fruit skins, Maker00038018 (*CLH1-like*) and Maker00016354 (*KNOX2*) had up-regulated expression levels in WS fruit skins. The remaining 16 DEGs (Maker00001799 *CHS2*; Maker00014265 *4CL2*; Maker00007651 *CHLM*; Maker00016117 *POR*; Maker00008808 *CAO*; Maker00003033 *SGR*; Maker00008858 *SGR-like*; Maker00022517 *CAB*; Maker00008553 *ARR17-like*; Maker00001025 *peroxidase 2-like*; Maker00017083 *PsaE*; Maker00014866 *PsbW*; Maker00033993 *HEMA1*; Maker00013112 *CRD1*; Maker00000841 *POR-like*; and Maker00022071 (*CAB*) had down-regulated expression levels in WS fruit skins (Fig. 8). The pearson correlation coefficient was 0.58 between the results of RNA-Seq and qRT-PCR validation. Thus, the qRT-PCR and RNA-Seq data were generally consistent.

### Subcellular localization analyses of selected proteins

**Figure 9:**
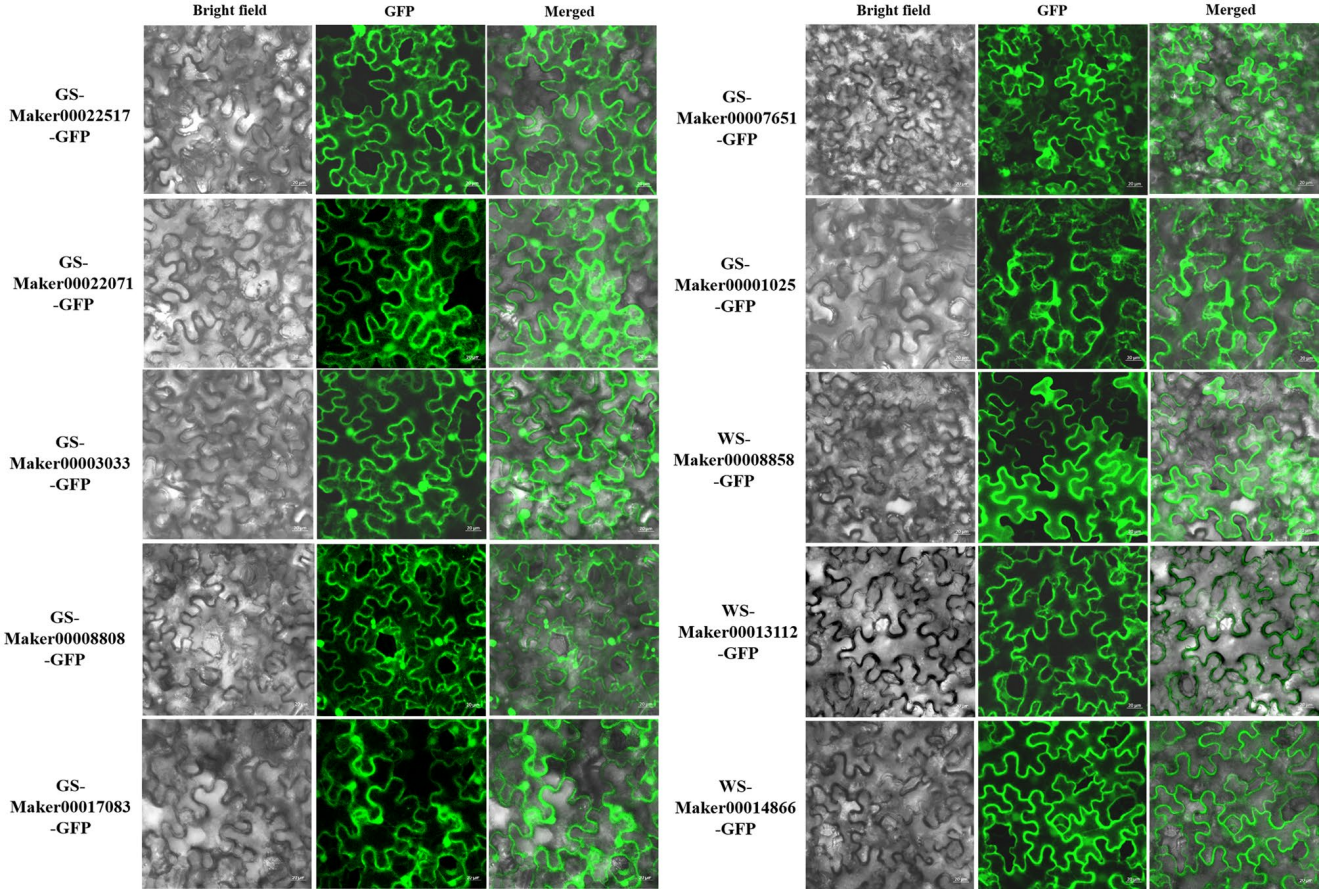
Subcellular localization analyses of selected proteins. Green fluorescent protein (GFP)-fusion proteins were transiently expressed in tobacco leaf epidermal cells after 48 h of incubation and the GFP signal was detected under a fluorescence microscope. The bright-field, fluorescence and merged images were shown for each protein.

The subcellular localization of ten selected proteins from GS and WS were analyzed by transient expression of the green fluorescent protein (GFP) fusion proteins in tobacco leaf epidermal cells. As shown in Fig. 9, six proteins from GS, including Maker00022517, Maker00022071, Maker00003033, Maker00008808, Maker00017083 and Maker00007651, were localized in the nucleus and cytoplasm, while the Maker00001025 protein from GS was localized in the nucleus, cytoplasm and endoplasmic reticulum. Moreover, three proteins from WS, including Maker00008858, Maker00013112 and Maker00014866 were localized in the nucleus and cytoplasm.

## Discussion

Researchers have focused on fruit skin color-related traits and revealed the associated genes and potential molecular mechanisms in cucumber^10–13^, tomato^14–16^, and potato^24^. However, sponge gourd fruit skin coloration remains relatively unexplored. This study involved an integrated investigation of the transcriptome, sRNAome, and degradome of two sponge gourd lines that differed in terms of their fruit skin colors (i.e., white and green). The generated data revealed several DE-miRNAs and DEGs involved in chlorophyll and flavonoid metabolism or chloroplast development and protection that influence sponge gourd fruit skin coloration.

The chlorophyll biosynthetic genes *HEMA*, *CHLM*, *CRD1*, *POR*, and *CAO* were expressed at lower levels in WS fruit skins than in GS fruit skins. Chlorophyll biosynthesis is tightly regulated by *HEMA*; this gene encodes a glutamyl-tRNA reductase, which is the initial enzyme of the rate-limiting step involving the synthesis of 5-aminolevulinic acid (ALA)^43^. In rice, *YGL18*, which encodes a magnesium protoporphyrin IX methyltransferase (CHLM), is essential for light-related chlorophyll synthesis and light intensity-associated plant growth^44^. The rice *PGL* gene encoding chlorophyllide *a* oxygenase 1 (CAO1) is mainly expressed in the chlorenchyma and is activated in the light-dependent chlorophyll synthesis process; compared with wild-type plants, *pgl* mutant plants have a lower chlorophyll content and a disordered thylakoid ultrastructure^45^. In the current study, the WS fruit skins had lower chlorophyll contents and altered chloroplast ultrastructures compared with the GS fruit skins. The down-regulated expression of these biosynthetic genes may help to explain the inhibited chlorophyll biosynthesis in WS fruit skins.

The expression of *SGR* and *CLH1*, which are involved in chlorophyll degradation, was also down-regulated in WS fruit skins. The *SGR* gene was initially identified in pea as a key regulator of chlorophyll degradation that is responsible for Mendel’s green cotyledon trait^46^. Mutations to STAY-GREEN-encoding homologs are responsible for the green flesh and chlorophyll retainer phenotypes of tomato and pepper^47^. Chlorophyllase (CLH) is the first enzyme in the chlorophyll catabolic pathway^48^. Earlier research identified CLH1 as a chlorophyll dephytylase involved in PSII repair in Arabidopsis^49^. The observed down-regulation in *SGR* and *CLH1* expression in WS fruit skins suggests chlorophyll degradation is also impaired in WS sponge gourd fruit skins.

In this study, DEGs associated with chloroplast development, including those encoding BEL1-like homeodomain protein, homeobox knotted-1-like protein, ARF, and ARR17-like protein, were identified. In ripening tomato fruits, *BEL1-LIKE HOMEODOMAIN4* influences chlorophyll accumulation, chloroplast development, cell wall metabolism, and carotenoid accumulation^50^. In the WS fruit skins, the Maker00014487 (BEL1-like homeodomain protein 7) expression level was up-regulated, in contrast to the down-regulated expression of the remaining four *BEL1-like* genes. The *KNOX* genes *TKN4* and *TKN2* function upstream of *GOLDEN2-LIKE 2* (*SlGLK2*) and the related gene *SlAPRR2-LIKE* to influence various chloroplast developmental processes in tomato fruits^17^. In this study, six *KNOX* genes and one *ARR17-like* gene were expressed at lower levels in WS fruit skins than in GS fruit skins. Previous studies demonstrated that SlARF4 negatively regulates chlorophyll accumulation in tomato fruits^19,20^, whereas SlARF10 positively regulates chlorophyll accumulation by activating *SlGLK1* expression^51^*.* Four ARF-encoding genes, *ARF3*, *ARF4*, *ARF6*, and *ARF8*, were more highly expressed in fruit skins of WS than in GS, whereas the opposite expression pattern was observed for the *ARF19-like* gene. These findings suggest *BEL1-like* and *KNOX* genes may enhance chlorophyll accumulation and chloroplast development in sponge gourd, whereas *ARF3*, *ARF4*, *ARF6*, and *ARF8* have the opposite effects.

In present study, the miR156, miR159, miR172, miR319, and miR396 family members were mostly up-regulated, whereas the miR166, miR167, miR171, miR390, and miR393 family members were mostly down-regulated in WS fruit skins (Table S5). Previous investigations determined miR156 is associated with anthocyanin accumulation in the pear fruit peel and in blueberry^22,23^. Additionally, miR396, miR156, miR171, and miR319 are reportedly associated with chlorophyll metabolism in plants^23,52–54^. For example, transgenic creeping bentgrass overexpressing *Osa-miR396c* (i.e., a rice miRNA396 gene) develops abnormally and accumulates more chlorophyll than the wild-type control^52^. The overexpression of the blueberry gene *VcMIR156a* in tomato enhances anthocyanin biosynthesis and chlorophyll degradation in the stem by altering pigment-associated gene expression, while also altering the chloroplast ultrastructure^23^. In Arabidopsis, the miR171–*SCL* module is critical for mediating GA–DELLA signaling during the coordinated regulation of chlorophyll biosynthesis^53^. Transgenic tomato plants overexpressing wild tomato (*Solanum habrochaites*) miRNA319d (sha-miR319d) exhibit enhanced stress tolerance and have high chlorophyll contents^54^. The miRNAs identified in the current study, including miR396, miR156, miR171, and miR319, are potential regulators of sponge gourd fruit skin coloration.

Eight DE-miRNAs targeting eight DEGs with opposite expression level and associated with chlorophyll binding and chloroplast development in sponge gourd were detected (Table 5). For example, the down-regulated miRNAs aly-miR167d-3p_2ss9CT20GA and cme-miR167d targeted the up-regulated Maker00008400 (*ARF8*) gene. More specifically, miR167, which is an important regulator of auxin-mediated development, reportedly targets the members of a large family of transcription factors that modulate gene expression in response to auxin (i.e., *ARF4*, *ARF6*, and *ARF8*)^55,56^. Earlier investigations confirmed *ARF4* controls fruit chloroplast development or chlorophyll biosynthesis in tomato^19,20^. In tobacco, NtARF8 regulates *ANS* and *DFR* expression in an *NtTTG2*-dependent manner, thereby contributing to anthocyanin production and flower coloration^57^. However, the involvement of miR167 and its target gene *ARF8* in chloroplast development or chlorophyll biosynthesis remains unknown.

Six miRNAs targeting seven DEGs with opposite expression level and associated with flavonoid synthesis in sponge gourd were identified (Table 6). For example, cme-miR396a negatively targeted *bHLH143*, cme-miR159a_R-1 negatively targeted *bHLH51*, cme-MIR319b-p5 negatively targeted *MYB*, cme-miR319c_R + 2_1ss20TC negatively targeted *CHS2*, and cme-miR396b and han-miR3630-3p_L-3 negatively targeted *4CL2*. The bHLH proteins form one of the largest transcription factor families in plants. The anthocyanin biosynthesis-related SGIIIf *bHLH* genes have been identified in horticultural crops, including those encoding bHLH3 and bHLH64 transcription factors in apple and pear^58,59^. The R2R3-MYB transcription factor genes *SlAN2-like* and *BrPAP1a* encode regulators of anthocyanin production in tomato and turnip^60,61^. Additionally, *CHS* and *4CL* are structural genes involved in anthocyanin biosynthesis. Accordingly, these miRNAs and their targets regulate flavonoid synthesis to determine sponge gourd fruit skin coloration.

## Conclusion

This study presented an integrated analysis of the transcriptome, sRNAome, and degradome between two materials of sponge gourd with distinct fruit skin colors to elucidate the genes, miRNAs and their network regulating fruit skin coloration. The crucial genes involved in chlorophyll metabolism, chloroplast development and chloroplast protection, including *HEMA*, *CHLM*, *CRD1*, *POR*, *CAO*, *CLH*, *SGR*, *CAB*, *BEL1-like*, *KNAT* and *ARF*, were identified. Moreover, the miR156, miR159, miR166, miR167, miR172, miR393 and their target genes involved in chlorophyll metabolism, chloroplast development and chloroplast protection were obtained. Additionally, the miR159, miR166, miR169, miR319, miR390, miR396 and their targets *CHS*, *4CL*, *bHLH* and *MYB* involved in flavonoid biosynthesis regulatory network were identified. These results provided the molecular mechanism of fruit skin coloration at the levels of transcriptome, sRNAome and degradome, and would lay foundation for further validation of key genes and miRNAs regulating fruit skin coloration in sponge gourd.

## Supplementary Information

Supplementary Information 1.

Supplementary Information 2.

Supplementary Information 3.

Supplementary Information 4.

Supplementary Information 5.

Supplementary Information 6.

Supplementary Information 7.

Supplementary Information 8.

## Data Availability

Raw sequencing data of transcriptome can be accessed through the GSA of National Genomics Data Center (NGDC) (https://ngdc.cncb.ac.cn/) with the accession number CRA005331.
