# Supplementary material for: Integrated analysis of the transcriptome, sRNAome, and degradome reveals the network regulating fruit skin coloration in sponge gourd (Luffa cylindrica)
Source: Sci Rep. 2022 Feb 28;12:3338. doi: 10.1038/s41598-022-07431-w (PMC8885689; doi:10.1038/s41598-022-07431-w)
Supplement: Supplementary file 8 — Supplementary Information 8. [file 41598_2022_7431_MOESM8_ESM.docx]

**Supplementary information**

**Manuscript title:** Integrated analysis of the transcriptome, sRNAome, and degradome reveals the network regulating fruit skin coloration in sponge gourd (*Luffa cylindrica*)

**Abstract:** Sponge gourd fruit skin color is an important quality-related trait because it substantially influences consumer preferences. However, little is known about the miRNAs and genes regulating sponge gourd fruit skin coloration. This study involved an integrated analysis of the transcriptome, sRNAome, and degradome of sponge gourd fruit skins with green skin (GS) and white skin (WS). A total of 4,331 genes were differentially expressed between the GS and WS, with 2,442 down-regulated and 1,889 up-regulated genes in WS. The crucial genes involved in chlorophyll metabolism, chloroplast development, and chloroplast protection were identified (e.g., *HEMA*, *CHLM*, *CRD1*, *POR*, *CAO*, *CLH*, *SGR*, *CAB*, *BEL1-like*, *KNAT*, *ARF*, and peroxidase genes). Additionally, 167 differentially expressed miRNAs were identified, with 70 up-regulated and 97 down-regulated miRNAs in WS. Degradome sequencing identified 125 differentially expressed miRNAs and their 521 differentially expressed target genes. The miR156, miR159, miR166, miR167, miR172, and miR393 targeted the genes involved in chlorophyll metabolism, chloroplast development, and chloroplast protection. Moreover, a flavonoid biosynthesis regulatory network was established involving miR159, miR166, miR169, miR319, miR390, miR396, and their targets *CHS*, *4CL*, *bHLH*, and *MYB*. The qRT-PCR data for the differentially expressed genes were generally consistent with the transcriptome results. Subcellular localization analysis of selected proteins revealed their locations in different cellular compartments, including nucleus, cytoplasm and endoplasmic reticulum. The study findings revealed the important miRNAs, their target genes, and the regulatory network controlling fruit skin coloration in sponge gourd.

**Keywords:** sponge gourd; fruit skin color; multi-omics; chlorophyll; flavonoid

**Contributing author details**

**Author lists:** Yuyan Sun^#^, Huiqing Zhang^#^, Wenqi Dong, Shengmi He, Shuting Qiao, Xingjiang Qi, Qizan Hu*

**First author:** Dr. Yuyan Sun

Institute of Vegetables, Zhejiang Academy of Agricultural Sciences, Hangzhou 310021, China

Tel: +86 571 8698 2260

E-mail: sunyy@zaas.ac.cn

**Co-first author:** Miss Huiqing Zhang

Institute of Vegetables, Zhejiang Academy of Agricultural Sciences, Hangzhou 310021, China

Tel: +86 571 8698 2260

1. mail address: [zhq111925@126.com](mailto:zhq111925@126.com)

**Third author:** Prof. Wenqi Dong

Institute of Vegetables, Zhejiang Academy of Agricultural Sciences, Hangzhou 310021, China

Tel: +86 571 8672 8170

E-mail address: [dwq9516@sina.com](mailto:dwq9516@sina.com)

**Fourth author:** Prof. Shengmi He

Institute of Vegetables, Zhejiang Academy of Agricultural Sciences, Hangzhou 310021, China

Tel: +86 571 8640 4322

E-mail address: hesm@zaas.ac.cn

**Fifth author:** Miss Shuting Qiao

Institute of Vegetables, Zhejiang Academy of Agricultural Sciences, Hangzhou 310021, China

Tel: +86 571 8698 2260

E-mail address: [qst20210306@126.com](mailto:qst20210306@126.com)

**Sixth author:** Prof. Xingjiang Qi

Institute of Vegetables, Zhejiang Academy of Agricultural Sciences, Hangzhou 310021, China

Tel: +86 571 8698 2260

E-mail address: qixj@zaas.ac.cn

***Corresponding author:** Prof. Qizan Hu

Institute of Vegetables, Zhejiang Academy of Agricultural Sciences, Hangzhou 310021, China

Tel: +86 571 8640 6758

1. mail address: huqz@zaas.ac.cn

**Legend of supplementary tables**

Table S1. Primers used for qRT-PCR validation of selected genes.

Table S2. Primers used for subcellular localization analyses of selected proteins.

Table S3. Genes detected as expressed at least one library and DEGs in WS and GS.

Table S4. DEGs encoding transcription factors in WS and GS.

Table S5. miRNAs, pre-miRNAs and DE-miRNAs identification in WS and GS.

Table S6. Target genes predicted for miRNAs using degradome sequencing technology.

Table S7. Network of DE-miRNAs and their DE-targets.
